# Elephantiasis Nostras Verrucosa

**Published:** 2012-10-12

**Authors:** Rafi Fredman, Mayer Tenenhaus

**Affiliations:** ^a^Sackler School of Medicine, Tel Aviv, Israel; ^b^Division of Plastic and Reconstructive Surgery, UC San Diego Medical Center, San Diego, Calif.

**Figure:**
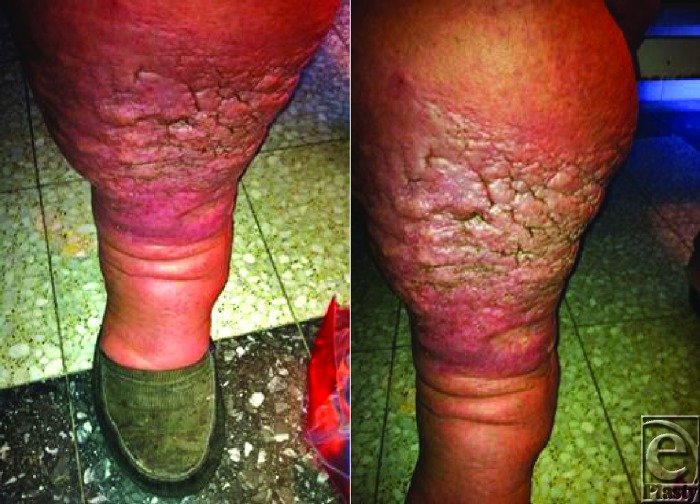


## DESCRIPTION

A 53-year-old woman presented to the hospital complaining of severe pain in her right leg for the past several days. She has a 5-year history of lymphedema in her right leg, as well as morbid obesity, recurrent cellulitis, erysipelas, and osteomyelitis. She has never suffered from filariasis and has no family history of familial lymphedema. Her temperature on admission was 100.9°F (38.3°C). Examination revealed a significantly enlarged, nonpitting, malodorous right leg with hyperpigmented, cobblestone-like lesions extending from the malleolus to several inches below the knee. The surrounding area was erythematous, warm to the touch, and tender.

## QUESTIONS

1. **What is elephantiasis?**
2. **What are the causes of lymphedema?**
3. **How does chronic lymphedema progress to elephantiasis nostras verrucosa (ENV)?**
4. **How does ENV present?**
5. **Discuss the differential diagnosis of ENV?**
6. **What therapeutic options are available to treat ENV?**

## DISCUSSION

Elephantiasis is thought to develop as the result of chronic lymphedema. Depending upon chronicity and severity, these patients often present with grossly enlarged and disfigured extremities characterized by a thickened and fibrotic appearing skin. Lymphedema is categorized into primary and secondary etiologies. Primary lymphedema can be caused by congenital agenesis, hypoplasia, or obstruction of lymphatic vessels, while secondary lymphedema results from a disruption or obstruction of previously normal lymphatic vessels.^1^ Worldwide, secondary lymphedema is most often caused by filariasis. In Western countries, causes include repeated streptococcal lymphangitis and erysipelas, malignancy, trauma, prior surgery, radiation therapy, chronic venous stasis, scleroderma, and obesity.^2^ Elephantiasis nostras verrucosa (ENV) is uncommon, arising in the setting of chronic nonfilarial lymphedema caused by bacterial or noninfectious lymphatic obstruction.

The prolonged accumulation of protein-rich fluid in the interstitium in patients with chronic lymphedema is thought to lead to a chronic inflammatory state with the accumulation of fibroblasts, adipocytes, and keratinocytes, transforming the initially soft but swollen tissue into hard fibrotic tissue with a thickened skin.^3^ Fibrosis of the dermis and subcutaneous tissue develops along with cutaneous changes of hyperkeratosis, papillomatosis, verrucous hyperplasia, and adipocyte proliferation.^4^ The International Society of Lymphology categorizes the progression of lymphedema into 4 stages (0-3) with lymphostatic elephantiasis representing the most severe progression of chronic lymphedema.^5^

Elephantiasis nostras verrucosa presents as a grossly enlarged and disfigured appendage, most commonly of the lower extremities and feet, with a cobblestone or mossy appearance. The skin feels “woody,” the edema is nonpitting, and does not resolve upon elevation of the extremity. The initial characteristic cobblestone appearance progresses to a verrucous and mossy appearance. With time, ulceration and crusting may complicate and the lesion eventually becomes chronically colonized by bacteria or fungi and emits a fetid odor. Lymph stasis attenuates local immune function, destabilizing wound development and predisposing the region to infection; thus, patients often present complaining of pain due to recurrent episodes of cellulitis, as was the case with our patient.^6^

The differential diagnosis of ENV is broad and includes lymphatic filariasis, chromoblastomycosis, lipedema, lipodermatosclerosis, papillomatosis cutis carcinoides, popular mucinosis, pretibial myxedema, and Stewart Treves syndrome.^7^ The diagnosis can often be elicited on the basis of history and physical examination and radiological imaging is rarely necessitated. A biopsy is important because of the risk of associated malignancy. 6^,^^8^ Characteristic histological findings include pseudoepitheliomatous hyperplasia, dilated lymphatic channels, widened tissue spaces, and extensive fibrous tissue hyperplasia within the dermis, subcutaneous tissue, and lymphatic vessel walls.^9^

Elephantiasis nostras verrucosa can be very difficult to treat, and comprehensive, long-term studies on the management of elephantiasis are unfortunately lacking. There is no standard of care in patients with ENV and the clinician is left with a handful of case reports and literature reviews to guide management.^9^ As always, the most important initial task is to investigate the underlying cause of lymphedema, which should be treated as best as possible. The initial therapy should be directed at alleviating lymphedema with massage, multilayer inelastic lymphedema bandaging, and compression stockings after confirming adequate vascular perfusion and control of any infection.^3^ More intensive therapy includes decongestive lymphatic therapy, which is a form of complex physical therapy that includes skin hygiene, limb compression, and exercise.^10^ Success has also been reported with external sequential pneumatic compression devices.^11^ Interestingly, liposuction has been advocated in less complicated presentations.^10^ Pharmacological intervention with oral or topical retinoids has been successfully employed to treat ENV and can be considered as an adjunct to physiotherapy.^12^ For cases that have not responded well to conservative or medical treatment, surgical intervention should be attempted. Surgical debridement has been reported to successfully reduce verrucous lesions in several case reports.^13^^-^15 It is important to note that surgical debulking does not address the underlying lymphatic pathology; nevertheless, it provides increased comfort by removing redundant skin and subcutaneous tissue. Several case reports have noted success in treating lymphedema with various microsurgical procedures. Although rarely used in the United States, both lymphaticovenular anastomosis as well as free muscle flap transfer have been used to resolve lymphedema in patients with obstructive lymphedema.^16^^,^^17^ Other treatments such as topical keratolytics (eg, salicylic acid) as well as deodorant powders to manage odor are commonly used. Limb amputation can be considered for ENV refractory to other treatment.
